# Mental Health Inequalities During COVID-19 Outbreak: The Role of Financial Insecurity and Attentional Control

**DOI:** 10.5334/pb.1064

**Published:** 2021-11-12

**Authors:** Nele Claes, Annique Smeding, Arnaud Carré

**Affiliations:** 1 Univ. Savoie Mont Blanc, Univ Grenoble Alpes, LIP/PC2S, F-73000, Chambéry, France

**Keywords:** Socioeconomic status, mental health inequalities, lockdown, COVID-19, financial insecurity, attentional control

## Abstract

The COVID-19 pandemic and subsequent lockdowns negatively impacted the mental health of populations. This impact is not equally distributed and increases existing mental health inequalities. Indeed, government restrictions and the economic consequences of the pandemic affect more the less educated and less wealthy people. However, psychological processes implicated in this increase of mental health inequalities during the COVID-19 pandemic remain unexplored. The present study (N=591) tested the role of financial insecurity and attentional control in the relation between socioeconomic status and mental health, along with the influence of trait anxiety. Based on Structural Equation Modelling, findings showed a mediation effect of financial insecurity, but not of attentional control, in the relationship between socioeconomic status and mental health. In addition, exploratory analyses suggested that financial insecurity also mediated the effect of attentional control on mental health. Results of the present research point at the importance of understanding psychological processes implicated in the effect of economic crises on mental health inequalities.

## Introduction

Since the end of 2019, the novel Coronavirus has given rise to a global and worldwide crisis. In France, as in many European countries, the COVID-19 pandemic has considerably impacted our daily life since spring 2020. Government restrictions to contain the spread of the virus included social distancing, closures of non-essential businesses, self-isolation, quarantine, and lockdown. Very quickly after, these measures resulted in a large slowdown of the economy (Baldwin et al., 2020). These restrictions and the associated economic downturn have manifested in unintended psychological consequences, such as a decrease of mental health (e.g., Kumar & Nayar, 2020; Shim, 2020; Torales et al., 2020). Mental health, as defined by the World Health Organization (WHO) refers to a state of well-being in its broad sense acceptation, an effective functioning of the individuals, and an effective functioning of a community (2004). This conception of mental health thus not solely includes the absence of mental illness, but also the presence of a flourishing life (i.e., high levels of well-being and optimal functioning, Keyes, 2005). In addition, in view of the strong associations of mental health issues with somatic health and general adaptation of individuals, WHO proposes that it is not conceivable to consider good health without mental health (Prince et al., 2007). The decrease of mental health due to government restrictions seems to be a consequence of quarantine and social isolation resulting in increased anxiety and depressive disorders (Wang et al., 2020; Wathelet et al., 2020), suicidal behaviours, or insomnia (Hao et al., 2020). These negative effects of the crisis on mental health are consequences of the pandemic context and lockdowns, considered as a traumatic event (Ettman et al., 2020, see Arora et al., 2020 for a review). As observed during previous epidemics (Brooks et al., 2020), the present crisis and associated stressors have already impaired mental health condition, increased rates of depression and anxiety disorders in the general population, as compared to before the onset of the pandemic (e.g., Ettman et al., 2020; Torales et al., 2020; Veer et al., 2021; Wang et al., 2020). However, although the general population reported a decrease in well-being, the increase of mental disorders seems particularly true for health workers and groups with pre-existing risks (e.g., patients with pre-existing psychiatric disorders or lower income individuals, Ettman et al., 2020; see Vindegaard & Benros, 2020 for literature review). However, few research focused on the potential psychological processes involved in the mental health consequences of lockdown. We suggest that social perception of the pandemic context may differ between low and high socioeconomic status (SES) individuals, which may increase mental health inequalities.^1^ In this research, we test a comprehensive model of mental health inequalities during France’s first lockdown including attentional control, financial insecurity, and trait anxiety.

### Financial Stressors and Mental Health

One major explanation of mental health consequences of the COVID-19 pandemic is the increase of stressors due to pandemics and quarantines (Brooks et al., 2020). To date, according to literature, several candidates can explain the relation between financial stress and mental illness, especially associated factors such as previous financial resources (before crisis), ability to meet expenses, long working time, gender or young children at home. For COVID-patients, there is an increase of stressors, such as financial loss due to the disease or disease-related stigma. For the general population, there are new stressors in daily life (e.g., fear of getting infected, inadequate information, reduced social interactions) (Blustein et al., 2020; Restubog et al., 2020; Rudenstine et al., 2021), along with an increase of financial stressors provoked by the economic recession during the lockdown. After a negative appraisal by individuals, these stressors become an economic stress, hence negatively impacting psychological functioning and increasing mental disorders (Frasquilho et al., 2016; Madianos et al., 2011; Probst et al., 2018). For instance, these stressors were shown to lead to more financial insecurity (Sinclair & Cheung, 2016), more future-related and economic uncertainty (e.g., job insecurity, economic hardship) which, in turn, resulted in poorer mental health (Dohrenwend, 2000; Wright et al., 2016). Given this, it is not surprising that previous research has found a link, in an economic crisis context, between financial stressors and the risk of psychological distress and depression, as shown for the Great Recession of 2008 (e.g., Kiernan, 2019; Zavras et al., 2013; see Frasquilho et al., 2016 for a review). As the pandemic has fuelled social and economic threats, one promising avenue to understand the influence of the pandemic on mental health is to focus on psychological processes related to how individuals process threats, including environmental ones.

### Attentional Control and Mental Health

One candidate process to account for the effect of the pandemic on mental health is attentional control. Attentional control usually refers to how individuals regulate attention allocation and is composed of two factors: focalisation of attention when facing distractors, and shifting attention between several competing tasks (Anderson, 2002; Judah et al., 2014). Poor attentional control is associated with poor regulation of attentional biases to threat, which are a central component of anxiety (see Bar-Haim et al., 2007 for a meta-analysis). The function of attentional biases is to maintain cognitive resources to threat and can be considered as an adaptive process by an increase of reactivity (Conejero & Rueda, 2018). However, this kind of information processing can become non-adaptive when attentional control is less efficient. For instance, when the threat information is not an objective threat (e.g., a common and non-venomous spider, a harmless puppy), or when the threat information is no longer relevant for the task (i.e., updating function and monitoring of information). Attentional control is a protective factor of maladaptive psychological outcomes and an aetiological factor of common mental disorders (i.e., depression and anxiety disorders; Eisenberg et al., 2010). Previous studies have shown that low attentional control leads to depression (Hsu et al., 2015), more worry, rumination and negative thinking (Armstrong et al., 2011; Cox et al., 2018). In the case of the COVID-19 pandemic, having efficient attentional control may serve as a protective factor when facing negative thinking and rumination about stressors, especially during lockdowns. Reversely, having less efficient attentional control should represent a factor of vulnerability which, in turn, leads to poorer mental health. In the present pandemic context, Jun et al. (2021) suggested that the crisis and the restricted financial resources have decreased attentional performance. Bardeen et al. (2021) had recently shown that individuals with less efficient attentional control had a stronger association between COVID-19 stress and generalised anxiety. Authors suggested that individuals who experienced high levels of stress due to the pandemic may have difficulties to disengage attention of their worries, particularly if they have poorer attentional control. We suggest that the role of attentional control in impairments lived by individuals do not refer to poor performance in attentional control *per se*, but to difficulties in regulating several social and affective constraints.

### Mental Health Inequalities during COVID-19

Furthermore, environmental factors such as socio-demographic characteristics have affected mental health outcomes during the crisis (Husky et al., 2020; Jeong et al., 2016; Math et al., 2008; Perrin et al., 2009) and have increased existing mental health inequalities. To account for these inequalities, the literature assumed a direct consequence of disparities in financial resources, which then decreases access to care for low SES individuals (Garrison & Rodgers, 2019). In addition, indirect consequences such as environmental constraints – including less material resources and limited social support (Kröger et al., 2015) – are assumed to impair cognitive processes and behaviours of low SES individuals, which in turn decrease mental health (Darin-Mattsson et al., 2018; Fors et al., 2009). In the pandemic context, vulnerability to poorer mental health caused by social isolation, the increase of stressors, and financial insecurity due to lockdowns are not equally distributed in the population (Witteveen & Velthorst, 2020). The most economically disadvantaged and stressed groups were those least able to isolate themselves to reduce the spread of the virus (Corburn et al., 2020) and were often infected (Schröder et al., 2020). In addition, economic recessions impact low SES individuals more than high SES ones (Jenkins et al., 2012; Smeeding et al., 2011). In the present crisis, low SES lose more often their jobs due to lockdowns – as there are more home working possibilities for higher income and higher qualified jobs – (Schröder et al., 2020), and have poorer housing conditions (Cheung & Ip, 2020).

These factors increase mental health inequalities. Prior research on the effects of restrictive government measures have also shown that low SES feel more disease-anxiety (i.e., anxiety of catching and transmitting the virus) and consequences-anxiety (i.e., anxiety regarding the consequences of lockdown) (McElroy et al., 2020). Lower educated individuals reported also more depressive symptoms (Gao et al., 2020; Mazza et al., 2020; Olagoke et al., 2020; Witteveen & Velthorst, 2020) and anxiety (Gao et al., 2020; Witteveen & Velthorst, 2020). In addition, higher income students (Rudenstine et al., 2021), and students with steady family income (Kontoangelos et al., 2020) reported lower anxiety. Amerio et al., (2020) have also shown that poor housing conditions during the first lockdown, such as smaller housing, lower indoor quality, and no outdoor space, were associated with more anxious and depressive symptoms. A study conducted in a French students’ sample showed that living alone in a residence far from family or having access to a garden played a differential effect on the way the lockdown was experienced (Husky et al., 2020). It was also significantly associated with poorer mental health outcomes such as higher levels of anxiety or higher stress levels related to the financial level (Husky et al., 2020). Although most research suggests that low SES represents a risk factor in terms of mental health in times of crisis, some studies found that high SES individuals reported more anxiety and depressive symptoms (Moghanibashi-Mansourieh, 2020; Wang et al., 2020) or did not find an effect of SES on anxiety during the lockdown (Mazza et al., 2020; Wang et al., 2020). Thus, although results regarding these effects of SES are not completely conclusive, most evidence goes in favour of a negative relation between SES and mental health outcomes, which we also expect to observe in the present study. However, as these aforementioned studies on mental health inequalities did not investigate potential processes implicated in these inequalities, examining some of them as they pertain to the lockdown during the COVID-19 pandemic seems paramount to tailor future interventions.

We already detailed why attentional control represents a good candidate for such a process account. In addition, given our focus on mental health inequalities and the specificities of lockdown, financial insecurity should also be considered. Particularly, as financial insecurity is a risk factor in the development of anxiety disorders and depression (Wright et al., 2016), we hypothesise that low SES individuals should have more financial stressors and less efficient attentional control than high SES, which should result in higher levels of perceived financial insecurity, and eventually in poorer mental health. Investigating this set of relations in a comprehensive model should steer a processual understanding of mental health inequalities during the lockdown, a timely contribution in times of crisis.

## Research overview

**Figure 1:**
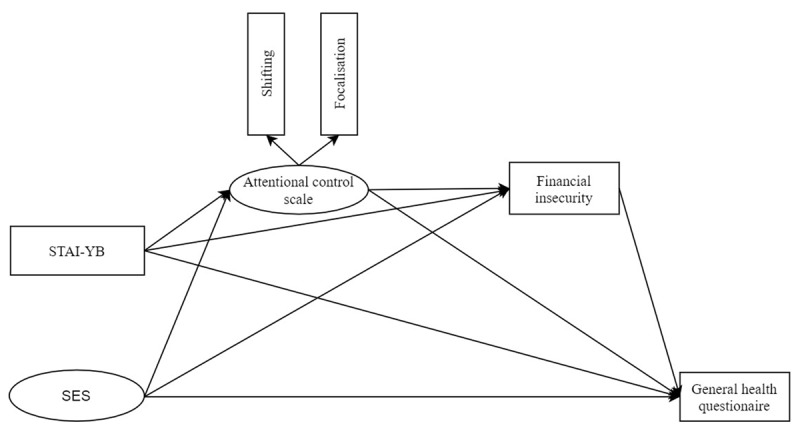
Theoretical model tested in the study. *Note*: The model tests the influences of socioeconomic status indicators measured by home possessions (SES) on attentional control pooled in a latent variable, financial insecurity, and mental health. Attentional control influences financial insecurity and mental health. The model also takes into account the effect of trait anxiety on attentional control, financial insecurity, and mental health.

As low SES individuals are more likely to be infected (Wachtler et al., 2020 for a literature review) and more likely to be financially insecure due to lockdown (Schröder et al., 2020), we hypothesise that in the current crisis, mental health consequences are SES-dependent. As past research did not investigate psychological processes that may be implicated in these mental health inequalities, we purpose here to test the mediating role of attentional control. Indeed, although the link between attentional control and maladaptive psychological outcomes is well studied, determinants of the efficiency of attentional control are not well known. On one hand, we retain trait anxiety – conceived of as an individual difference variable – as it is related to attentional control (Derryberry & Reed, 2002), intolerance to uncertainty (e.g., Pawluk & Koerner, 2016), and SES (Croizet et al., 2017). On the other hand, in addition to a stable individual difference antecedent like trait anxiety, our focus is on psychosocial antecedents involved in the onset and maintenance of several mental disorders (Eaton et al., 2009) and which may also influence attentional control. Supporting the relevance of psychosocial antecedents, recent research shows that higher SES is associated with higher attentional control (Conejero & Rueda, 2018). We therefore expect to observe a positive relation between SES and attentional control. In addition, given the importance of financial stressors in this type of crisis, having efficient attentional control may serve as a protective factor when facing negative thinking and rumination about these stressors. We therefore expect attentional control to mediate the effect of financial stressors on perceived financial insecurity. The target outcome variable is a measure of mental health developed for the general population, which assesses mental distress and is predictive of psychiatric disorders (Aalto et al., 2012; Gnambs & Staufenbiel, 2018). It seeks to detect somatic symptoms as well as socialisation or emotional disorders (including anxious or depressive symptoms). To summarise, we hypothesise that low SES individuals are more likely to report (i) less attentional control, (ii) more financial insecurity, and (iii) poorer mental health, even when controlling for trait anxiety. These hypotheses are tested using Structural Equation Modelling (SEM) and ***Figure 1*** summarises the theoretical model of the present study. The target period covers in part France’s first lockdown (from April 7th to May 7th, 2020).

## Method

### Participants

The sample was recruited via social media advertisement and is composed of 1123 French-speaking participants from France and Belgium. Participants were excluded if they did not complete the whole survey (517) or did not consent to the collection of their responses after debriefing (12). These exclusion criteria left a final sample of 591 participants (*M =* 39.18 years old, *SD =* 17.49), essentially women (82%). Based on actual recommendations, the minimal acceptable sample size was 220 participants, which thus served as the lower boundary (Kline, 2015). All participants completed an online consent form. The study was preregistered on OSF (*https://osf.io/9mcep/*).

### Measures

#### Mental health

We used the General Health Questionnaire (GHQ-28; Goldberg & Hillier, 1979; validated in French by Pariente et al., 1992) to measure participants’ mental health. Participants completed the questionnaire with the instructions to evaluate their mental health in general over the past few weeks. The GHQ comprises 28 items from 0 (Better than usual) to 3 (Much worse than usual) or from 0 (Not at all) to 3 (Much more than usual), depending on items. Higher mean scores reflect a poorer mental health (M = 31.58, SD = 16.23, α = .94). Descriptive statistics for subscales and by gender are provided for all measures in Supplementary Materials (Table S1).

#### Trait anxiety

Trait anxiety was assessed using the part B of the State and Trait Anxiety Inventory (STAI-YB) questionnaire (Bruchon-Schweitzer & Paulhan, 1993; Spielberger, 1983). Trait anxiety represents individuals’ propensity to respond in an anxious way in general. This questionnaire comprises 20 items (e.g., “I have disturbing thoughts”), with responses on a 4-point Likert scale from 1 (Almost never) to 4 (Almost always) (M = 46.69, SD = 9.82, α = .88). Higher mean scores reflect higher levels of trait anxiety.

#### Attentional control abilities

We used the French version of the Attentional Control Scale (ACS, Judah et al., 2014, Leleu et al., in press), which includes 20 items scored from 1 (*Almost never*) to 4 (*Almost ever*). The questionnaire evaluates two independent dimensions which represent focusing (9 items; e.g., “It’s very hard for me to concentrate on a difficult task when there are noises around”, M = 22.73, SD = 5.08, α = .79) and shifting (11 items; e.g., “It’s easy for me to alternate between two different tasks”, M = 30.67, SD = 5.19, α = .73). Higher mean scores reflect more attentional control abilities (i.e., focusing and shifting).

#### Objective socioeconomic status

##### Home possessions

We adapted the material deprivation questionnaire of the British Household Panel Survey (wave 17, Taylor et al., 2018) which includes two scales. First, participants were asked whether they possessed several material possessions at home (e.g., dishwasher, television) (1 = *Yes*, 0 = *No*). Scores were calculated as the sum of the eighteen home possessions (M = 13.92, SD = 2.70). Second, participants were asked whether they were financially able to have some activities, such as buying some clothes or having a decent decoration (1 = *Yes*, 0 = *No*). The score was the sum of the nine activities (M = 6.89, SD = 2.31).

##### Classical measures of socioeconomic status

Participants completed the three classical socioeconomic indicators: occupation, educational level, and income. First, occupation is composed of six subcategories, divided into two larger categories which will be used in analyses: low SES (coded -0.5; n = 257) composed of “Employee”, “Worker”, and “Unemployed” subcategories, and high SES (coded 0.5, n = 204) composed of “Artisans, shopkeepers, CEOs”, “Executives and intellectual professions” and “Intermediate professions subcategories”. Students were coded 0 (n = 130). Second, education level, composed of six ranks (from 1 = *no diploma* to 6 = *Master degree or more*, was categorised in two categories: low SES (coded –0.5; n = 194), and high SES for participants holding a college degree or more (coded 0.5, n = 397). Based on the literature (Goudeau et al., 2017), students were categorised based on mother’s education level.^2^ Third, annual household income, comprising six levels from 1 (less than 12.000 euros) to 6 (more than 60.000 euros) (M = 3.16, SD = 1.60).

#### Financial Insecurity

We adapted the financial insecurity scale (Price et al., 2002) to the lockdown context. The scale was composed of three items (e.g., “To what extent do you think the consequences of outbreak will create financial hardship for you and your family?”) from 1 = *Not at all difficult* to 10 = *Very difficult* (M = 8.28, SD = 4.57, α = .91).^3^

## Results

### Analytical procedure

We used the Lavaan package (version 0.6–5, Rosseel, 2014) to run SEM. Because item distributions did not follow a normal distribution, we used the MLR estimator (Finney & DiStefano, 2006). We used the model generalisation approach proposed by Jöreskorg and Sörbom (1993, cited by Gana & Broc, 2018). This approach is used when the fit indices of the theoretical model are not acceptable, and the model is modified and retested (Gana & Broc, 2018). The acceptability of fit indices was evaluated following current recommendations: CFI ≥ .90; TLI ≥ .90; RMSEA ≤.08; SRMR < .08; and χ^2^/df <3 (Kline, 2015).

### Confirmatory analyses

**Figure 2:**
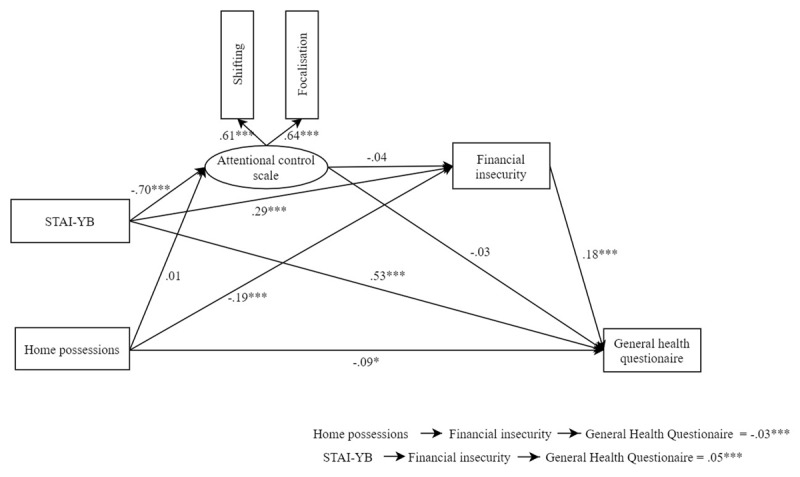
Summary of Model A2. *Note*: The figure includes the direct effects, as well as the main indirect effects of Model A2. *** *p* < .001; ** *p* < .01; * *p* < .05.

The test of the theoretical model (Model A1) had one negative variance, which may signal model misspecification (Chen et al., 2001). The second dimension of home possessions had a negative variance, probably due to the non-normality of distribution, thus we tested the model without this dimension (Model A2). The model with this modification (Model A2) was better than Model A1 (Δ χ^2^(5) = 96.36, *p* < .001) and showed excellent fit indices (see ***Table 1*** for all fit indices). As predicted, the model showed that home possessions negatively predicted mental health (*B* = –.09, 95% CI [–.16, –.02], *p* = .014) and financial insecurity (*B* = –.19, 95% CI [–.26, –.11], *p* < .001), but did not predict attentional control (*B* = .01, 95% CI [–.12, .12], *p* = .978). In addition, financial insecurity positively predicted poor mental health (*B* = .18, 95% CI [.11, .25], *p* < .001). Contrary to hypotheses, attentional control did not predict financial insecurity (*B* = –.04, 95% CI [–.14, .06], *p* = .405) or mental health (*B* = –.03, 95% CI [–.12, .05], *p* = .438). Trait anxiety negatively predicted attentional control (*B* = –.7, 95% CI [–.84, –.57], *p* < .001), and positively predicted financial insecurity (*B* = .29, 95% CI [.18, .39], *p* < .001) and poor mental health (*B* = .53, 95% CI [.44, .63], *p* < .001). For all estimates of Model A, see ***Table 2***. The indirect effects show that financial insecurity mediated the effect of home possessions on global health (indirect effect: *B* = –.03, 95% CI [–.05, –.01], *p* < .001) and the effect of trait anxiety on mental health (indirect effect: *B* = .05, 95% CI [.03, .08], *p* < .001). However, attentional control did not mediate the effect of trait anxiety on financial insecurity (indirect effect: *B* = .03, 95% CI [–.04, .10], *p* = .404) and on mental health (indirect effect: *B* = .02, 95% CI [–.04, .08], *p* = .438). ***Figure 2*** displays results for this Model A2.

**Table 1:** Fit indices for all models.


	χ^2^	χ^2^/df	CFI	TLI	RMSEA [CI-90%]	SRMR

Model A1	105.86	13.23	.917	.782	.149 [.125, .176]	.120

Model A2	8.53	2.84	.994	.970	.055 [.012, .100]	.013

Model B	99.65	7.12	.920	.839	.110 [.091, .131]	.110

Model B1 – Income	9.38	3.13	.991	.957	.071 [.028, .119]	.015

Model B2 – Occupation	5.83	1.94	.997	.984	.04 [.001, .088]	.011

Model B3 – Education	5.78	1.93	.997	.985	.039 [.001, .088]	.011

Model C	2.26	1.13	.997	.997	.014 [.001, .08]	.011


*Note*: χ^2^ = Chi square; χ^2^/df = Chi square/degree of freedom; CFI = Comparative Fit Index; TLI = Tucker-Lewis Index; RMSEA = Root Mean Square Error of Approximation; SRMR = Standardized Root Mean Square Residual.

**Table 2:** Estimates of direct and indirect effects of Model A2.


	ESTIMATE	95% CI	Z-VALUE	P-VALUE

Latent variable				

A.C. → ACS Foc.	0.64	[0.56, 0.73]	14.97	<.001

A.C. → ACS Shif.	0.61	[0.54, 0.69]	16.06	<.001

Direct effects				

Home Poss. → A.C.	0.02	[–0.12, 0.12]	0.03	.978

STAI-YB → A.C.	–0.70	[–0.84, –0.57]	–10.01	<.001

A.C. → GHQ	–0.03	[–0.12, 0.05]	–0.78	.438

Home Poss.→ GHQ	–0.09	[–0.16, –0.02]	–2.46	.014

STAI-YB → GHQ	0.53	[0.44, 0.63]	11.36	<.001

F.I. → GHQ	0.18	[0.11, 0.25]	5.16	<.001

Home Poss. → F.I.	–0.19	[–0.26, –0.11]	–4.72	<.001

STAI-YB → F.I.	0.29	[0.18, 0.39]	5.42	<.001

A.C. → F.I.	–0.04	[–0.14, 0.06]	–0.83	.405

Indirect effects				

STAI-YB→ A.C. → GHQ	0.02	[–0.04, 0.08]	0.78	.438

STAI-YB→ F.I. → GHQ	0.05	[0.03, 0.08]	3.91	<.001

STAI-YB→ A.C. → FI	0.03	[–0.04, 0.1]	0.84	.404

Home Poss. → A.C. → GHQ	0.01	[0.00, 0.00]	–0.03	.979

Home Poss. → F.I. → GHQ	–0.03	[–0.05, –0.01]	–3.40	.001

Home Poss. → A.C. → FI	0.00	[–0.01, 0.01]	–0.03	.979

A.C. → F.I. → GHQ	–0.01	[–0.03, 0.01]	–0.81	.419


*Note*: A.C. = Attentional Control; ACS Foc = Focalisation’s dimension of the ACS; ACS Shif = Shifting dimension of the ACS; Home Poss = Home Possessions; STAI = State Trait Anxiety Inventory (Y-B version); GHQ = General Health Questionnaire; F.I. = Financial Insecurity.

In addition to the focal Model A2 including home possessions, we also tested the model with classical indicators of SES (Model B). However, Model A2 was better than Model B (Δ χ^2^(11) = 90.57, *p* < .001), which had also worse fit indices.

### Exploratory analyses

As preregistered, we tested Model B with the classical indicator of SES (i.e., income, education, occupation). The model had no satisfactory fit indices (see ***Table 1***). Because SES is multidimensional, we also tested each indicator of SES separately (Models B1, B2, B3). These models were better than model B (Δ χ^2^(11) = 90.27, *p* < .001 for income, Δ χ^2^(11) = 93.87, *p* < .001 for education, Δ χ^2^(11) = 93.82, p < .001 for occupation) and better than model A2, except for the model with income. Estimators were equivalent to model A2 except for the direct effect of SES on mental health (non-significant here) and on attentional control, which was predicted by income (*B* = .10, 95% CI [.02, .17], *p* = .013). All estimators are available in Supplementary Materials (Table S3 and Table S4).^4^

Finally, we tested a model (Model C) without the STAI-YB to test a confounding effect (MacKinnon et al., 2000) of trait anxiety on the effects of attentional control. This model had excellent fit indices, better than the other models (see ***Table 1***). Conversely to Model A2, and in alignment with hypotheses, attentional control predicted global health (*B* = –.32, 95% CI [–.41, –.23], *p* < .001) and financial insecurity (*B* = –.21, 95% CI [–.30, –.12], *p* < .001). However, home possessions did not predict attentional control (*B* = .03, 95% CI [–.07, .13], *p* = .507). This alternative model is presented in Supplementary Materials (Figure S1).

## Discussion

The present study aimed to test a comprehensive model of mental health inequalities during lockdown, including financial insecurity and attentional control. We hypothesised that low SES individuals were more likely to report (i) less attentional control, (ii) more financial insecurity, and (iii) poorer mental health. Regarding the mediation mechanism, we reasoned that because low-SES individuals should have lower attentional control to cope with stressors due to lockdown, they would feel more financial insecurity which should then be associated with poorer mental health.

Results partly confirmed this hypothesised process. First, the present results indicated that financial insecurity was associated with poorer mental health and mediated the effect of SES (i.e., home possessions). This finding is consistent with prior research indicating that the economic consequences of the COVID-19 pandemic are not equally distributed among social groups, neither are its mental health consequences (e.g., Witteveen & Velthorst, 2020). A recent longitudinal study confirmed a negative effect of pandemic on mental health and the increase of mental health inequalities (Ettman et al., 2020). The association between financial stressors and mental health may have increased with the pandemic context (Probst et al., 2018). These effects could be explained by environmental factors, in particular the increase of financial stressors due to lockdown and the associated perceived financial insecurity, whether actual (e.g., difficulty to make the ends meet because of salary decrease) or prospective (e.g., fear of job loss). The economic fallout due to the COVID-19 pandemic and the rapid increase of unemployment has probably increased stressors and perceived financial insecurity as suggested by Benach et al. (2014), and as found after the Great Recession of 2008 (Kopasker et al., 2018).

Second, contrary to hypotheses and despite good fit indices for the confirmatory model, results indicated that attentional control was not associated with financial insecurity, mental health, and SES. However, exploratory analyses (with Model C) suggested a confounding effect between trait anxiety and attentional control, due to a large negative correlation between these two variables (MacKinnon et al., 2000). Leaving out trait anxiety of the model led to observe the expected relations between attentional control, financial insecurity, and mental health, although home possessions remained unrelated to attentional control. According to attentional control theory, poorer attentional control leads to be more focalised on financial stressors and financial insecurity, a similar process to what has been found for uncertainty more generally in past research (Frings et al., 2019). To our knowledge, this study is the first to test the relation between attentional control and financial insecurity, but future studies are needed: first, to replicate results based on exploratory analyses in the present research and second, to better understand the relation between attentional control and financial insecurity.

Third, concerning the operationalisation of SES, we tested the model with several measures of SES (i.e., home possessions and classical indicators of SES; Models B). Exploratory analyses showed that effects of SES varied depending on the indicator. The model had better fit indices with home possessions than with classical indicators of SES, a result in line with prior research showing that housing size and access to outdoor space during lockdown could have positive effects on mental health (Amerio et al., 2020; Husky et al., 2020; Pouso et al., 2021). This highlights the importance of economic factors, including ownership and a kind of independence linked to housing, on the quality of life during an event such as the pandemic. Our study suggests that the economic dimension of SES, measured with income and home possessions (representing access to resources), had stronger associations with financial insecurity. In addition, only income was related to attentional control. This result is also in line with prior research suggesting that dimensions of SES (represented by indicators of SES) are not equally involved in psychological processes (Goudeau et al., 2017; Kraus et al., 2012). For example, Ettman et al., (2020) showed that income, but not education was associated with the increase of depressive symptoms during the lockdown. One potential explanation for this result is that income is more representative of the financial stressors than home possessions, which is in line with research indicating that financial concerns impede cognitive functions such as working memory (Mani et al., 2013) and decision making (Shah et al., 2012). As we did not include an objective measure of financial stressors (e.g., financial loss), future studies are needed to explore whether objective financial stressors are associated with attentional control, and subsequently with financial insecurity and mental health.

### Limitations and Perspectives

The study has some limitations. First, although we targeted a representative community sample, women and students were overrepresented. These groups were more impacted by lockdown, possibly resulting in more negative consequences on their mental health. Although results were similar when excluding students from analyses, future studies with a more representative sample are needed to confirm and generalise the present results. Second, we did not use sheer attentional checks in the study, because participation was voluntary and these checks may involve mistrust (Chandler et al., 2014). Participants were asked to report their seriousness after completion, but this does not guarantee only honest answers on this item. However, the fact that all participants who did not complete the whole survey were excluded certainly has the advantage of keeping only those who were most conscientious. Third, as our focus was on testing a comprehensive model and a possible process account of mental health disparities during the COVID-19 outbreak and resulting first lockdown, the present cross-sectional design was favoured. As a result, we cannot assume that the present findings are necessarily specific to the pandemic and its context, as we did not measure pandemic stressors or anxiety (e.g., McElroy et al., 2020), neither did we collect measures before the COVID-19 crisis or several months after the first lockdown. In fact, although highly relevant to the current crisis, we assume that the model tested in the present study is a comprehensive model of mental health inequalities which is particularly helpful to understand the increase of these inequalities during and after economic recessions, including the recession due to the COVID-19 pandemic. Future research may therefore aim to go a step further, for instance by assessing the causal relationships assumed in the model through longitudinal studies.

## Conclusion

As during prior recessions, the recession due to the COVID-19 pandemic could increase financial insecurity and increase mental health inequalities. The present research showed an important role of trait anxiety, conceived of as an individual difference variable and associated with SES. This factor may be relevant when screening for risk factors in mental health during crises because it is quite likely that mental health information is also linked to the fundamental question of access to appropriate care. The global nature of this health crisis, which has also become a social and economic crisis, indicates that a response that addresses all these dimensions is much needed. For example, school courses should offer a form of psychoeducation in mental health and its disorders. Among these, the accessibility of mental health practitioners (such as psychologists and psychiatrists) should be improved, be it by increasing dedicated staff in care structures or by covering their cost in private practices by health insurance and the government. Moreover, the acute phase of the present crisis, and its long-term consequences, tend to show that the disruption of mental health must be regarded as a significant threat. Community interventions may also address the mental health inequalities issue, but need more evidence-based studies (see McGrath et al., 2021 for a review). As suggested by Ridley et al. (2020), future interventions to limit the negative impact of the COVID-19 pandemic and its direct consequences are much needed, and financial insecurity seems to be an important variable to include as suggested by the present research.

## Additional File

The additional file for this article can be found as follows:

10.5334/pb.1064.s1 Supplementary Materials. Table S1. Means and standard deviation by gender. Tables S2. Means, standard deviation and correlations between subjective measure of SSS (financial insecurity, personal relative deprivation, and SSS). Table S3. Estimates of Model B. Table S4. Estimates of Models B1, B2, and B3 (classical indicators of SES). Table S5. Estimates of Model C (with the anxiety dimension of GHQ). Table S6. Estimates of Model A2 without students. Figure S1 Summary of Model D.

## Footnotes

The study of health inequalities, and more particularly of the link between socioeconomic status and mental health, commonly suggests that mental disorders are indicators impaired mental health. This is particularly the case when studying the direct or indirect effects of poverty. We suggest that the effect of SES should be observed on an overall mental health level.

Because students were forced in this category, we tested the model without them. Results were equivalent and are presented as Supplementary Materials (see Table S6).

We also included the Personal Relative Deprivation Scale (Callan et al., 2008) and the MacAthur scale (Adler et al., 2000), because we were interested in comparing models with subjective socioeconomic status (SSS), relative deprivation, and financial insecurity. To measure SSS, participants were instructed to evaluate their perceived social rank in society on a drawn ladder with 10 rungs (*M* = 6.82, *SD* = 1.82). To measure relative deprivation, participants answered how they agree/disagree with 7 sentences (“I feel at a disadvantage when I compare my possessions with those of others who are look like”, M = 13.67, SD = 4.74, α = .78). Correlations between these two variables and financial insecurity are presented in Supplementary Materials (Table S2). Results of these models, which are not developed further here, are available upon reasonable request.

It were model fit issues that drove model modifications and it is not appropriate in SEM to use Bonferroni corrections (or any other correction for multiple testing) because the estimations of models and parameter estimates are not equivalent to the general linear model approach (see Kline, 2015 for information on model estimation in SEM).
