# Supplementary material for: Mental Health Inequalities During COVID-19 Outbreak: The Role of Financial Insecurity and Attentional Control
Source: Psychol Belg. 2021 Nov 12;61(1):327–40. doi: 10.5334/pb.1064 (PMC8588930; doi:10.5334/pb.1064)
Supplement: Supplementary Materials. — Table S1. Means and standard deviation by gender. Tables S2. Means, standard deviation and correlations between subjective measure of SSS (financial insecurity, personal relative deprivation, and SSS). Table S3. Estimates of Model B. Table S4. Estimates of Models B1, B2, and B3 (classical indicators of SES). Table S5. Estimates of Model C (with the anxiety dimension of GHQ). Table S6. Estimates of Model A2 without students. Figure S1 Summary of Model D. [file pb-61-1-1064-s1.pdf]

"Mental Health Inequalities During COVID-19 Outbreak: The Role of Financial Insecurity and Attentional Control"

Supplementary Materials

Preregistration and Data are available on the following link:

https://osf.io/9mcep/

Table S1

Means and Standard Deviation by gender.

Variable	Women	Men	Statistic	*p*
Age	37.77 ± 16.58	46.14 ± 19.41	t(581) =4.45	<.001
Home possessions				
Scale 1	9.45 ± 2.40	9.24 ± 2.50	t(581) = -0.80	.422
Scale 2	6.77 ± 2.33	7.43 ± 2.10	t(581) = 2.59	.010
Income	3.10 ± 1.57	3.47 ± 1.68	t(581) = 2.05	.041
Educational level	0.16 ± 0.47	0.22 ± 0.45	t(581) = -1.15	.250
Occupation	- 0.08 ± 0.43	0.13 ± 0.46	t(581) = -4.25	<.001
GHQ	35.75 ± 15.94	26.62 ± 16.73	t(581) = 3.46	<.001
Subscale anxiety	9.72 ± 5.50	7.48 ± 5.19	t(581) = 3.74	<.001
Subscale depression	5.00 ± 5.17	3.96 ± 5.23	t(581) = 1.82	.069
Subscale somatic symptoms	8.39 ± 4.79	6.28 ± 4.62	t(581) = 4.02	<.001
Subscale social dysfunction	9.64 ± 3.89	8.90 ± 4.21	t(581) = 1.71	.087
ACS Foc.	22.50 ± 5.12	23.76 ± 4.88	t(581) = -2.26	.024
ACS Shif.	30.63 ± 5.21	30.93 ± 5.17	t(581) = -0.52	.604
Financial Insecurity	8.55 ± 4.63	6.99 ± 4.11	t(581) = 3.12	.002
STAI-YB	47.10 ± 9.89	44.60 ± 9.52	t(581) = 2.31	.021

Note. ACS Foc. = Focalisation' dimension of the Attentional Control Scale; ACS Shif = Shifting dimension of the Attentional Control Scale; STAI-YB = State Trait Anxiety Inventory (Y-B version); GHQ = General Health Questionnaire.

Table S2

Means, Standard Deviation and correlations of Financial Insecurity, Personal Relative Deprivation, and SSS

Variable	*M* ± *SD*	1	2
1. Financial Insecurity	8.28 ± 4.57		
2. Subjective SES	5.99 ± 1.81	-.56** [-.61, -.50]	
3. Personal Relative Deprivation Scale	13.44 ± 5.17	.46** [.39, .52]	-.44** [-.50, -.37]

Note. *M* and *SD* are used to represent mean and standard deviation, respectively. Values in square brackets indicate the 95% confidence interval for each correlation. The confidence interval is a plausible range of population correlations that could have caused the sample correlation (Cumming, 2014). * indicates $p < .05$. ** indicates $p < .01$.

Table S3

Estimates of Model B

Variable	*B*	CI [95%]	*z*	*p*
Latent variables				
A.C. → ACS Foc.	0.65	[0.56, 0.74]	14.97	<.001
A.C. → ACS Shif.	0.62	[0.54, 0.71]	16.06	<.001
SES → Income	0.78	[0.69, 87]	16.36	<.001
SES → Education	0.25	[0.20, 0.29]	10.46	<.001
SES → Occupation	0.20	[0.16, 24]	9.23	<.001
Direct effects				
SES → A.C.	0.16	[0.00, 0.31]	2.01	.045
STAI-YB → A.C.	- 0.64	[- 0.79, - 0.49]	- 0.84	<.001
A.C. → GHQ	- 0.04	[- 0.13, 0.05]	- 0.84	.400
SES → GHQ	- 0.05	[- 0.18, 0.07]	- 0.82	.411
STAI-YB → GHQ	0.54	[0.44, 0.64]	10.45	<.001
FI → GHQ	0.17	[0.06, 0.27]	3.017	.003
SES → FI	- 0.56	[- 0.66, - 0.46]	- 10.75	<.001
STAI-YB → FI	0.17	[0.07, 0.28]	3.17	.002
A.C. → FI	0.01	[- 0.10, 0.11]	0.12	.904

Note. A.C. = Attentional Control; ACS Foc = Focalisation' dimension of the ACS; ACS Shif = Shifting dimension of the ACS; SES = Socioeconomic status; STAI-YB = State Trait Anxiety Inventory (Y-B version); GHQ = General Health Questionnaire; F.I.= Financial Insecurity.

Table S4

	Model B: Income				Model B2: Education				Model B3: Occupation			
	B	CI [95%]	*z*	*p*	*B*	CI [95%]	*z*	*p*	*B*	CI [95%]	*z*	*p*
Latent variables												
A.C. → ACS Foc.	0.64	[0.55, 0.73]	14.01	<.001	0.64	[0.56, 0.72]	15.32	<.001	0.64	[0.56, 0.72]	15.21	<.001
A.C. → ACS Shif.	0.63	[0.55, 0.71]	15.00	<.001	0.61	[0.54, 0.69]	16.10	<.001	0.61	[0.54, 0.69]	16.13	<.001
Direct effects												
SES → A.C.	0.16	[0.03, 0.28]	2.48	.013	0.15	[- 0.08, 0.38]	1.25	0.21	0.01	[- 0.22, 0.24]	0.09	.932
STAI-YB → A.C.	- 0.63	[- 0.78, - 0.49]	- 8.45	<.001	-0.69	[- 0.83, - 0.55]	- 9.55	<.001	- 0.70	[- 0.84, - 0.57]	- 9.95	<.001
A.C. → GHQ	- 0.04	[- 0.13, 0.05]	- 0.85	.397	-0.03	[- 0.12, 0.05]	- 0.72	.472	- 0.03	[- 0.12, 0.05]	- 0.74	.457
SES → GHQ	- 0.04	[- 0.12, 0.05]	- 0.87	.383	-0.03	[- 0.17, 0.11]	- 0.40	.692	0.04	[- 0.11, 0.19]	0.49	.623
STAI-YB → GHQ	0.54	[0.44, 0.64]	10.48	<.001	0.54	[0.44, 0.63]	11.22	<.001	0.54	[0.44, 0.63]	11.19	<.001
FI → GHQ	0.18	[0.09, 0.27]	4.07	<.001	0.20	[0.13, 0.27]	5.47	<.001	0.20	[0.13, 0.27]	5.71	<.001
SES → FI	- 0.44	[- 0.52, - 0.36]	- 10.98	<.001	-0.59	[- 0.76, - 0.41]	- 6.61	<.001	- 0.34	[- 0.51, - 0.16]	- 3.78	<.001
STAI-YB → FI	0.18	[0.07, 0.29]	3.30	<.001	0.25	[0.14, 0.36]	4.53	<.001	0.27	[0.16, 0.38]	4.93	<.001
A.C. → FI	- 0.01	[- 0.12, 0.09]	- 0.23	.815	-0.02	[- 0.12, 0.08]	- 0.47	.636	- 0.04	[- 0.14, 0.06]	- 0.81	.418

Estimates of Models B1, B2, and B3 (classical indicators of SES)

Note. A.C. = Attentional Control; ACS Foc = Focalisation' dimension of the ACS; ACS Shif = Shifting dimension of the ACS; SES = indicator of SES, depending of model; STAI = State Trait Anxiety Inventory (Y-B version); GHQ = General Health Questionnaire; F.I.= Financial Insecurity.

Table S5

Estimates of Model C (with the anxiety dimension of GHQ)

	Estimate	CI [95%]	z-value	p-value
Latent variables				
A.C. → ACS Foc.	0.64	[0.56, 0.72]	14.90	<.001
A.C. → ACS Shif.	0.61	[0.54, 0.69]	16.02	<.001
Direct effects				
Home Poss. → ACS	0.01	[-0.12, 0.12]	0.03	.976
STAI-YB → ACS	-0.70	[-0.84, -0.57]	-10.01	<.001
ACS → Anxiety	-0.03	[-0.12, 0.07]	-0.55	.583
Home Poss. → Anxiety	-0.07	[-0.14, 0.01]	-1.81	.071
STAI-YB → Anxiety	0.48	[0.38, 0.57]	9.76	<.001
FI → Anxiety	0.13	[0.06, 0.21]	3.58	<.001
Home Poss. → FI	-0.19	[-0.26, -0.11]	-4.72	<.001
STAI-YB → FI	0.29	[0.18, 0.39]	5.42	<.001
ACS → FI	-0.04	[-0.14, 0.06]	-0.83	.407

Note. A.C. = Attentional Control; ACS Foc = Focalisation' dimension of the ACS; ACS Shif = Shifting dimension of the ACS; Home Poss = Home Possessions; STAI = State Trait Anxiety Inventory (Y-B version); Anxiety = Anxiety' dimension of GHQ; F.I.= Financial Insecurity.

Table S6.

Estimates of Model A2 without students

Variable	Estimate	CI [95%]	z-value	P(>|z|)
A.C. → ACS Foc.	0.61	[0.52, 0.70]	13.63	< .001
A. C. → ACS Shif.	0.68	[0.58, 0.77]	14.26	< .001
Home Poss. → A.C.	0.07	[-0.07, 0.20]	0.97	.332
STAI-YB → A.C.	-0.64	[-0.79, -0.49]	-8.41	< .001
A.C. → GHQ	-0.02	[-0.11, 0.08]	-0.40	.689
Home Poss.→ GHQ	-0.10	[-0.18, -0.03]	-2.65	.008
STAI-YB → GHQ	0.53	[0.43, 0.63]	10.64	< .001
FI → GHQ	0.17	[0.09, 0.25]	4.31	< .001
Home Poss. → FI	-0.15	[-0.24, -0.07]	-3.42	.001
STAI-YB → FI	0.25	[0.13, 0.36]	4.25	< .001
A.C. → FI	-0.11	[-0.23, 0.00]	-1.98	.048
STAI-YB→ A.C. → GHQ	0.01	[-0.05, 0.07]	0.40	.690
STAI-YB→ FI → GHQ	0.04	[0.02, 0.07]	3.14	.002
STAI-YB→ A.C. → FI	0.07	[0.00, 0.15]	1.96	.051
Home Poss. → A.C. → GHQ	0.00	[-0.01, 0.01]	-0.36	.719
Home Poss. → FI → GHQ	-0.03	[-0.05, -0.01]	-2.62	.009
Home Poss. → A.C. → FI	-0.01	[-0.03, 0.01]	-0.84	.403
A.C. → FI → GHQ	-0.02	[-0.04, 0.00]	-1.72	.086

Note. A.C. = Attentional Control; ACS Foc = Focalisation’ dimension of the ACS; ACS Shif = Shifting dimension of the ACS; Home Poss. = Home Possessions; STAI = State Trait Anxiety Inventory (Y-B version); GHQ = General Health Questionnaire; F.I.= Financial Insecurity. Fit indices of the model: CFI = .999; TLI = .995; RMSEA=.023[.000, 0.83]; SRMR =.01; and χ^2/df =1.234.

Figure S1

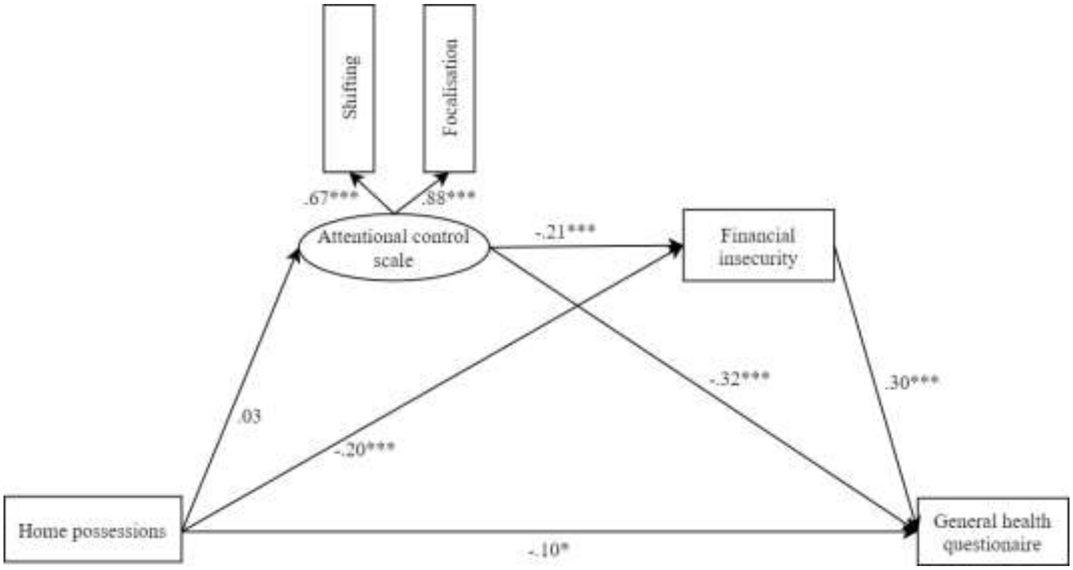

Summary of Model D

Note. The figure includes the direct effects of Model D. *** p<.001; ** p<.01; * p<.05.
